# Unexpected 16S rRNA heterogeneity in ‘Acetobacterium dehalogenans’ and reclassification as Acetobacterium malicum subsp. dehalogenans subsp. nov.

**DOI:** 10.1099/ijsem.0.006783

**Published:** 2025-05-08

**Authors:** Stefan Spring, Jacqueline Wolf, Sarah Kirstein, Cathrin Spröer, Boyke Bunk

**Affiliations:** 1 Department of Microorganisms, Leibniz Institute DSMZ - German Collection of Microorganisms and Cell Cultures, Braunschweig, Germany; 2 Department of Metabolomics and Services, Leibniz Institute DSMZ - German Collection of Microorganisms and Cell Cultures, Braunschweig, Germany; 3 Department Bioinformatics, Leibniz Institute DSMZ - German Collection of Microorganisms and Cell Cultures, Braunschweig, Germany

**Keywords:** evolution, homoacetogen, methylotroph, ribosome, SSU rRNA

## Abstract

Strain MC^T^ is a strictly anaerobic, homoacetogenic bacterium with the ability to utilize methyl chloride as the sole energy source. It was tentatively assigned to the genus *Acetobacterium* as ‘*Acetobacterium dehalogenans*’. Due to sequence ambiguities, it was not possible to determine the 16S rRNA gene sequence of this strain by direct sequencing of a PCR-amplified DNA segment. Whole-genome sequencing revealed significant heterogeneity amongst the five rRNA operons detected in this strain, with maximum sequence differences between the individual 16S rRNA genes exceeding 1.4%, compared to <0.8% in related species. Genome comparisons identified strain MC^T^ as most closely related to *Acetobacterium malicum* MuME1^T^, with a digital DNA–DNA hybridization value of 71.9% and an average nucleotide identity score of 96.59%, indicating that the strains belong to the same species. Both strains share the ability to utilize malate, a key feature of *A. malicum*, but differ in the utilization of methanol and glucose. Chemotaxonomic analyses also revealed distinct fatty acid and polar lipid patterns. Based on these findings, we propose the classification of strain ‘*Acetobacterium dehalogenans*’ MC^T^ (=DSM 11527^T^=NBRC 117038^T^) as *A. malicum* subsp. *dehalogenans* subsp. nov. This automatically establishes *A. malicum* subsp. *malicum* subsp. nov., with MuME1^T^ (=DSM 4132^T^=ATCC 51201^T^) as the type strain.

## Introduction

The genus *Acetobacterium* belongs to the family *Eubacteriaceae* in the class *Clostridia* and, at the time of writing, contains eight species with validly published names [1]. Representatives of this genus are strictly anaerobic, short rods that can grow autotrophically by oxidation of H_2_ and reduction of CO_2_ to acetate or chemoorganotrophically by homoacetogenic fermentation of reduced substrates [2]. In 1991, a homoacetogenic bacterium with the ability to utilize methyl chloride as the sole energy source was isolated [3] and later tentatively named ‘*Acetobacterium dehalogenans*’ [4]. A draft assembly of the genome of ‘*A. dehalogenans*’ strain MC^T^ consisting of 80 contigs was determined and deposited in NCBI GenBank under the accession AXAC00000000. The published draft genome contains only two truncated sequences of 16S rRNA genes with less than 400 nucleotides, which limits their use for phylogenetic analyses. Phylogenomic analyses based on average nucleotide identity (ANI), average amino acid identity and the Rnf multisubunit protein complex revealed a close relationship with *Acetobacterium malicum* at the species level [5]. A culture of ‘*A. dehalogenans*’ was deposited with the Leibniz Institute DSMZ in 1997 as DSM 11527^T^ and later subjected to a routine authenticity check by direct sequencing of the PCR-amplified 16S rRNA gene. It turned out that the chromatograms obtained by the Applied Biosystems 3500 Genetic Analyzer could not be evaluated due to many ambiguous peaks at the beginning of the 16S rRNA gene sequence. Therefore, the 16S rRNA genes were cloned, and several of the clones obtained were sequenced to verify the purity and authenticity of the culture. The cloned 16S rRNA genes revealed several divergent sequences, which prompted us to further investigate the RNA genes in this strain using genome sequencing.

## Methods

### Genome sequencing and analyses

Genomic DNA was isolated from cultures of DSM 2925^T^, DSM 11527^T^ and DSM 16427 grown to stationary phase under the cultivation conditions specified in the Media*Dive* database [6]. Genomic DNA was extracted from centrifuged cell pellets using the Epicentre-Lucigen MasterPure Gram-positive DNA Purification Kit (Biozym, Hessisch Oldendorf, Germany). Sequencing of the complete genome of ‘*A. dehalogenans*’ DSM 11527^T^ was performed using long-read PacBio sequencing technologies as described below. SMRTbell^®^ template library was prepared according to the instructions from Pacific Biosciences, Menlo Park, CA, USA, following the Procedure and Checklist – Preparing whole genome and metagenome libraries using SMRTbell^®^ prep kit 3.0. Briefly, for the preparation of 10 kb libraries, 2 µg of genomic DNA was sheared using the Megaruptor^®^ 3 from Diagenode, Denville, NJ, USA, according to the manufacturer’s instructions. DNA was end-repaired and ligated to barcoded adapters applying components from the SMRTbell^®^ prep kit 3.0 from Pacific Biosciences. Reactions were carried out according to the manufacturer’s instructions. Samples were pooled equimolarly. Conditions for annealing of sequencing primers and binding of polymerase to purified SMRTbell^®^ template were assessed according to the Sample Setup in SMRT^®^ Link, Pacific Biosciences. Libraries were sequenced on the PacBio Sequel IIe system (Pacific Biosciences) taking one 30 h movie per SMRT cell. A long-read genome assembly was performed with the ‘Microbial Assembly’ protocol included in SMRT Link version 11 using default parameters with the exception of the target genome size. The chromosomal contig was circularized; particularly, artificial redundancies at the ends of the contigs were removed and adjusted to *dnaA*. Redundancies were identified using blast, and replication genes were identified based on a Prokka annotation [7]. Circularization and rotation to the replication genes were performed using the genomecirculator.py tool (https://github.com/boykebunk/genomefinish).

In addition, complete genomes of the *Acetobacterium carbinolicum* strains DSM 2925^T^ and DSM 16427 were determined using the methods described above.

For comparative analyses, the complete genome sequence of *Acetobacterium woodii* DSM 1030^T^ (CP002987), *Acetobacterium wieringae* strain Y (CP087994) and *Acetobacterium* sp. KB1 (CP030040) and draft genomes of *A. malicum* DSM 4132^T^ (WJBE00000000), *A. wieringae* DSM 1911^T^ (LKEU00000000), *Acetobacterium paludosum* DSM 8237^T^ (WJBD00000000), *Acetobacterium fimetarium* DSM 8238^T^ (WJBC00000000), *Acetobacterium bakii* DSM 8239^T^ (LGYO00000000) and *A. tundrae* DSM 9173^T^ (RXYB00000000) were obtained from NCBI GenBank.

The characteristics and quality assessment of the three fully sequenced genomes, along with the draft genome of DSM 4132^T^, are presented in Table 1.

**Table 1.** Characteristics of genome sequences used in the phylogenomic analyses of the species *A. malicum* and *A. carbinolicum*. The genome sequence of *A. malicum* DSM 4132^T^ has been published previously [5]. The quality of the assembled genomes was assessed with CheckM2 [30]

	*A. malicum* DSM 4132^T^	‘*A. dehalogenans*’ DSM 11527^T^	*A. carbinolicum* DSM 2925^T^	*A. carbinolicum* DSM 16427
Sequencing technology	Illumina MiSeq	PacBio Sequel	PacBio Sequel	PacBio Sequel
Assembly method	SPAdes v. 3.7.0	SMRT Link v. 11.1.0	SMRT Link v. 13.1.0	SMRT Link v. 13.1.0
Assembly ID (GenBank)	GCA_014284495.1	GCA_047456285.1	GCA_047456275.1	GCA_047456265.2
Total genome size (bp)	4,077,227	4,140,558	4,094,424	4,148,202
No. of chromosomes	1	1	1	1
No. of plasmids	0	0	0	0
No. of contigs	71	1	1	1
G+C content (mol%)	43.5	43.8	42.4	42.3
Genome coverage	25.0×	45.0×	35.0×	33.0×
Completeness	100%	100%	99.98%	99.99%
Contamination	3.62%	0.53%	0.07%	0.96%
Coding density	0.876	0.871	0.875	0.875
Total no. of genes	3,855	3,839	3,820	3,851
- CDS	3,781	3,762	3,743	3,775
- rRNA	16	16	16	16
- tRNA	54	57	56	57
- ncRNA	1	4	5	3

Digital DNA–DNA hybridization (dDDH) values between genomes were calculated using the Type Strain Genome Server with the formula *d4*, provided by the DSMZ Digital Diversity hub (https://tygs.dsmz.de/ [8]). ANI values were calculated using the EZ BioCloud ANI calculator based on the OrthoANIu algorithm [9].

### Reconstruction of trees based on rRNA genes

Phylogenetic calculations based on 16S rRNA gene sequences were based on the alignment provided by the silva database SSU Ref NR 99 release 138.1 [10]. In addition, sets of five 16S rRNA gene sequences were extracted from the complete genomes of ‘*A. dehalogenans*’ DSM 11527^T^, *A. wieringae* strain Y and *A. carbinolicum* strains DSM 2925^T^ and DSM 16427. These sequences were then aligned with the arb-silva dataset. The alignment of 23S rRNA gene sequences for the reconstruction of phylogenetic trees was determined by the muscle web interface provided by EMBL-EBI [11]. The neighbour-joining algorithm included in the arb software package [12] with the correction of Felsenstein [13] was used for the reconstruction of phylogenetic trees of 16S and 23S rRNA gene nucleotide sequences.

### Reconstruction of whole-genome-based trees

The available genomes of the type strains of *Acetobacterium* were selected together with some additional reference strains and submitted to the GTDB-Tk version 2.3.2 for classification [14]. Phylogenomic trees were reconstructed by the IQ-TREE web server version 1.6.12 based on the concatenated amino acid alignment of 120 conserved bacterial proteins of the selected genomes generated by the CheckM application implemented in GTDB-Tk [15].

### Physiological tests and chemotaxonomy

Substrate utilization tests with the strains DSM 11527^T^ and DSM 4132^T^ were carried out in DSMZ medium 135, which was modified by omitting yeast extract and cysteine. The carbon source l-malate was used in a concentration of 10 mM. The growth response was recorded after three consecutive transfers in the same medium.

Biomass for chemotaxonomic analyses was harvested from cultures grown at 30 °C to stationary phase in DSMZ medium 135 (https://mediadive.dsmz.de/medium/135) modified by reducing the amount of d-fructose to 4.00 gl^−1^. The analyses of cellular fatty acids were done as described by Nouioui *et al*. [16]. Polar lipids were extracted from freeze-dried biomass using a modified Bligh and Dyer extraction [17]. Briefly, samples were extracted twice with methanol:dichloromethane (DCM):0.3% NaCl (2 : 1 : 0.8 v/v/v) by sonication for 10 min. The combined DCM phases were extracted twice with 0.3% NaCl and subsequently evaporated under a stream of nitrogen. Prior to analysis, the extracts were reconstituted in hexane/isopropanol/water (718 : 271 : 10 v/v/v) and filtered through a regenerated cellulose syringe filter (Minisart RC4; Sartorius, Göttingen, Germany). Intact polar lipids were analysed by HPLC-MS as described previously [18].

## Results and discussion

### Phylogenomic analysis of the genus *Acetobacterium*

**Fig. 1.**
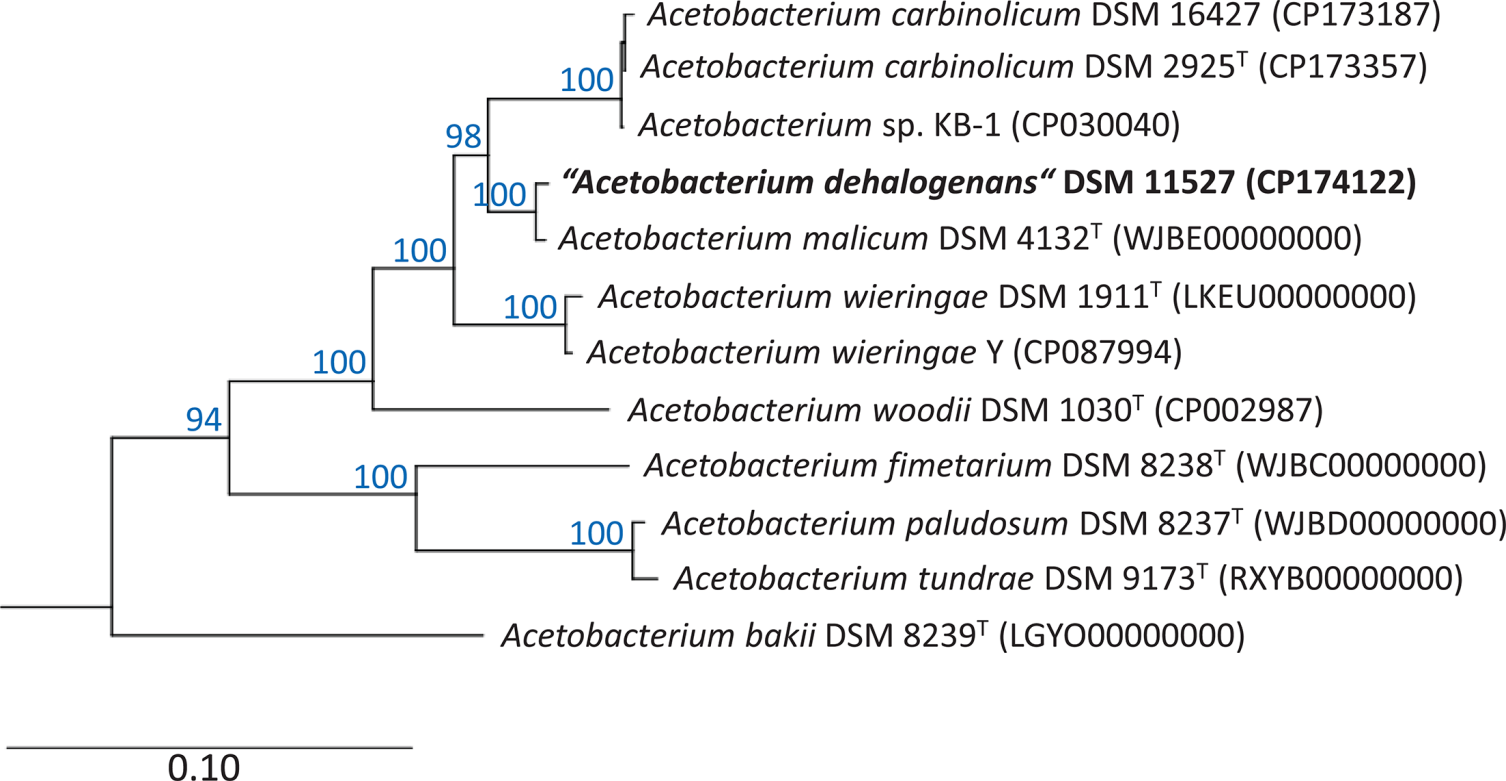
Phylogenomic tree showing the placement of ‘*A. dehalogenans*’ DSM 11527^T^ within the genus *Acetobacterium*. The shown consensus tree is based on trees reconstructed under the maximum-likelihood criterion with the model LG+F+I+G4 of protein evolution and ultrafast bootstrap analysis with a maximum of 1,000 iterations. The genome of *Eubacterium limosum* strain ATCC 8486^T^ (CP019962) was used as an outgroup (not shown). Accession numbers are given in parentheses. Only bootstrap support values above 80% are shown at the respective nodes. The scale bar indicates the expected number of substitutions per site.

A phylogenetic tree reconstructed based on the aligned amino acid sequences of 120 concatenated conserved bacterial proteins revealed the position of ‘*A. dehalogenans*’ within the genus *Acetobacterium*. The closest related species is clearly *A. malicum* with 100% bootstrap support in the consensus tree shown in Fig. 1. dDDH values and ANI values between both genomes were 71.9% and 96.59 %, respectively. This indicates that both strains belong to the same species, as values of 70% dDDH and 95% ANI are generally recognized as threshold values for the delimitation of species [19]. Recently, a 79% dDDH threshold has been introduced for the definition of subspecies based on comparative genome analyses of *Escherichia coli* strains and representatives of several other genera [20], whilst a threshold of 98% ANI has been established for the differentiation of *Salmonella* subspecies [21]. Therefore, it seems justified to define ‘*A. dehalogenans*’ as a subspecies of *A. malicum* and to propose the names *A. malicum* subsp. *dehalogenans* subsp. nov. and *A. malicum* subsp. *malicum* subsp. nov. accordingly.

The comparative genome analyses revealed also that the unclassified strain *Acetobacterium* sp. KB-1 belongs to the species *A. carbinolicum* as the genome of the type strain DSM 2925^T^ shares a dDDH value of 90.9% and an ANI value of 98.80% with strain KB-1. On the other hand, the previously proposed subspecies ‘*A. carbinolicum* subsp. *kysingense*’ [22] could not be confirmed, as the dDDH and ANI values between the genomes of the *A. carbinolicum* strains DSM 16427 and DSM 2925^T^ were 87.4% and 98.47 %, respectively, and thus within the range of a subspecies.

### Heterogeneity of rRNA genes in *Acetobacterium* strains

Five different rRNA operons were identified in all *Acetobacterium* strains with an available complete genome sequence. The least divergence was found in the 16S rRNA genes between the operons of the *A. carbinolicum* strain KB-1 with a 16S rRNA gene sequence identity of not less than 99.87%. For all strains of this species, the difference in the 16S rRNA sequence is less than 0.8%. Nearly identical 16S rRNA genes were also detected in the type strain of *A. woodii* DSM 1030^T^ with identity values of 99.67% or above, so far representing the *Acetobacterium* species with the lowest heterogeneity of rRNA genes. On the other hand, the identity values of 16S rRNA genes between different species of the genus *Acetobacterium* were as low as 96.99% (Fig. S1, available in the online Supplementary Material). This demonstrates that the mechanisms which control the concerted evolution of multiple rRNA genes within a species are principally active in most members of the genus *Acetobacterium*. The underlying molecular processes that prevent a genetic drift of multiple rRNA operons are, however, not well understood, but gene conversion by non-reciprocal homologous recombination [23] and horizontal gene transfer (HGT) within species [24] seem to play an important role.

A notable exception represents the 16S rRNA genes of ‘*A. dehalogenans*’ DSM 11527^T^, some of which show considerable sequence differences with identity values of only 98.56%, which is below the widely accepted threshold of 98.65% for species demarcation [25]. The dynamic evolution of rRNA operons appears to affect only this strain within the genus *Acetobacterium* and was not detected in the most closely related strain DSM 4132^T^. Unfortunately, only a draft genome of *A. malicum* DSM 4132^T^ is available, which contains a single copy of a 16S rRNA gene, but in contrast to DSM 11527^T^, a clean sequence could be obtained by direct sequencing of a PCR-amplified 16S rRNA gene.

**Fig. 2.**
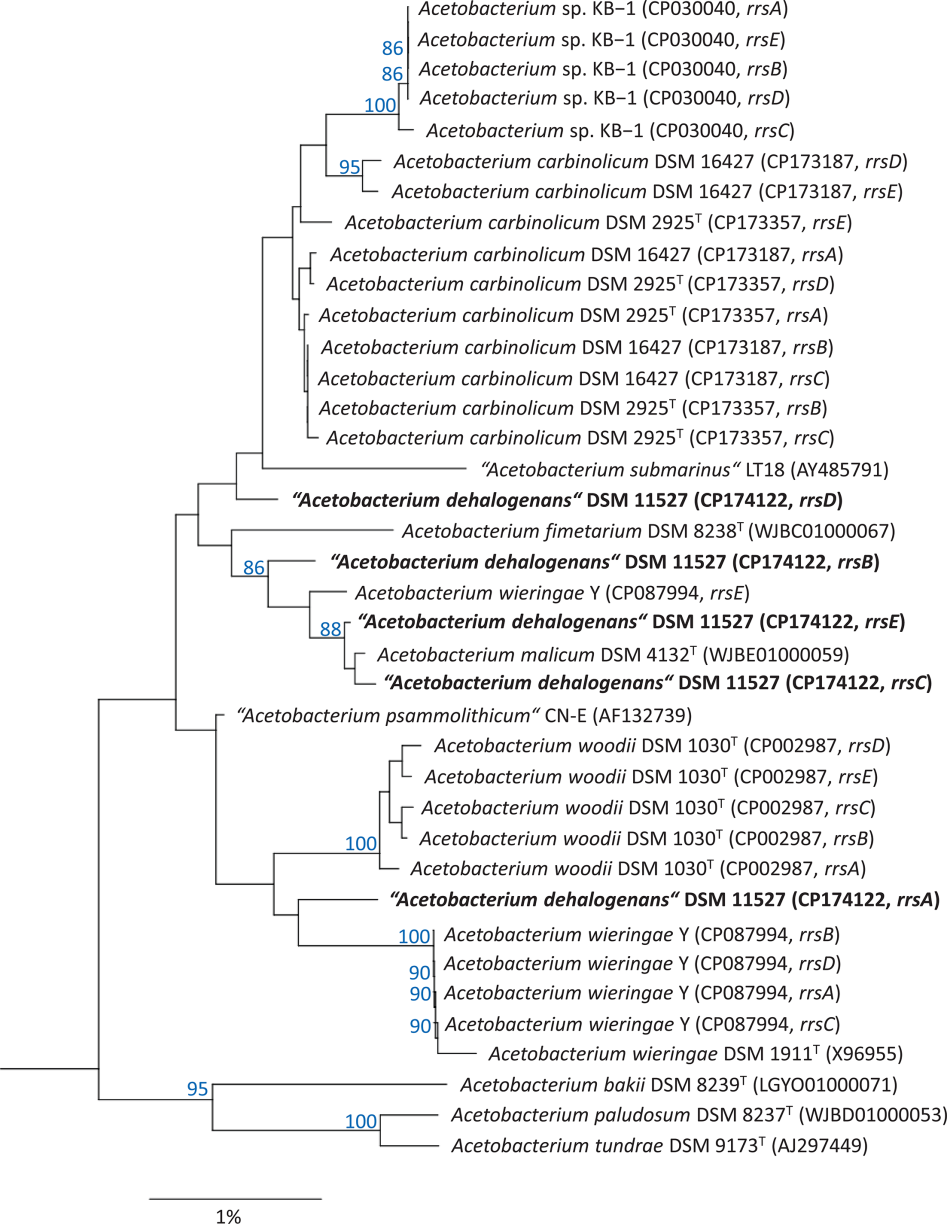
Phylogenetic tree of the genus *Acetobacterium* based on 16S rRNA gene sequences. The tree topology was reconstructed using the neighbour-joining algorithm and rooted using the 16S rRNA gene sequence of *E. limosum* JCM 6421^T^ (AB595134, not shown). Accession numbers are given in parentheses. Support of a distinct branching based on bootstrap analysis with 1,000 iterations is indicated by confidence values in percent of 100. Only values above 80% are shown at the respective nodes. Scale bar, 1% estimated sequence divergence.

**Fig. 3.**
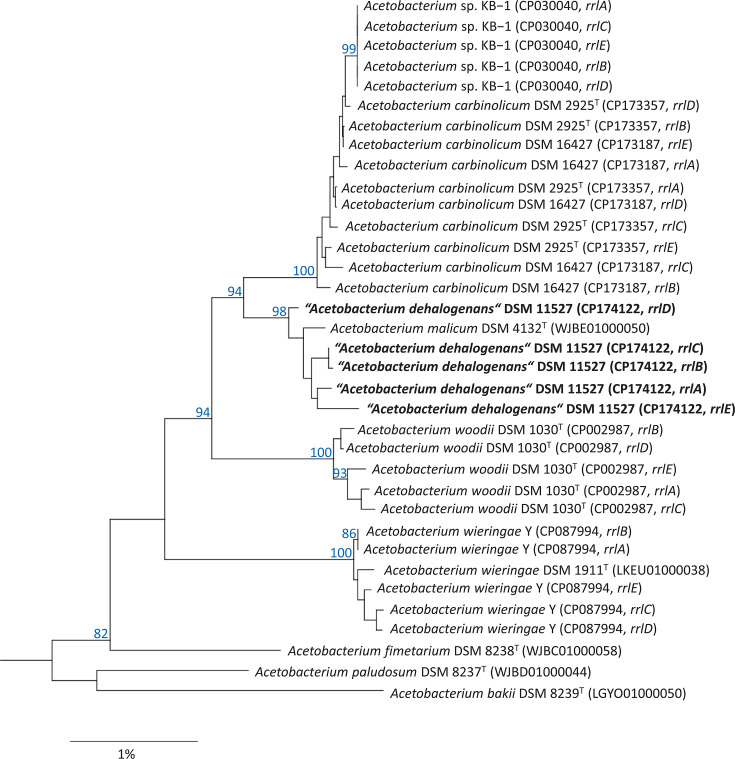
Phylogenetic tree of the genus *Acetobacterium* based on 23S rRNA gene sequences. The tree topology was reconstructed using the neighbour-joining algorithm and rooted using the 23S rRNA gene sequence of *E. limosum* ATCC 8486^T^ (CP019962 : 501184–498320, not shown). Accession numbers are given in parentheses. Support of a distinct branching based on bootstrap analysis with 1,000 iterations is indicated by confidence values in percent of 100. Only values above 80% are shown at the respective nodes. Scale bar, 1% estimated sequence divergence.

A comparison of the divergent 16S rRNA genes in strain DSM 11527^T^ shows that variations mainly affect regions V1 and V6 of the rRNA gene, which is in line with an earlier study based on more than 1,800 bacterial genomes [26]. More specifically, most variable nucleotide positions were located in the helices 6 (pos. 61–106), 10 (pos. 183–193) and 37 (pos. 1006–1023, *E. coli* numbering). Apparently, the rRNA genes in this strain evolve dynamically, i.e. without the constraints that normally prevent the genetic drift of multiple copies of rRNA genes in a species. A phylogenetic tree based on 16S rRNA genes seems to confirm this assumption, as the different copies of the rRNA genes of strain DSM 11527^T^ do not form a monophyletic group (Fig. 2), but rather cluster with the species *A. malicum* (*rrsB*, *rrsC* and *rrsE*), *A. carbinolicum* (*rrsD*) and *A. wieringae* (*rrsA*). In contrast, the tree based on 23S rRNA genes shows that the sequences of DSM 11527^T^ form a monospecific lineage together with the sequence of *A. malicum* DSM 4132^T^ that is supported by high bootstrap support (Fig. 3). This would be consistent with the idea that, in bacteria, intrageneric HGT and recombination mainly affect the 16S rRNA genes, but rarely other core genes of the cellular protein synthesis apparatus [24]. However, it could also be that the entire RNA operons are affected by the same genetic drift caused by mutations, but the positions of 23S rRNA genes in the tree are less sensitive to sequence divergence due to their higher phylogenetic information content [27].

In any case, it is puzzling why, only in strain DSM 11527^T^, the rRNA genes are not subject to coherent evolution compared to other strains of the genus *Acetobacterium*. We hypothesize that the differences may be due to a variation in the DNA repair and homologous recombination systems expressed in the strains of this genus.

### Phenotypic comparisons

Although the proposed type strain of ‘*A. dehalogenans*’ was described, in some detail, already in 1991 [3], so far, no formal proposal of a species name has been published. Based on the comparative genome analysis, the strain ‘*A. dehalogenans*’ MC^T^ is most closely related to the type strain of *A. malicum*. Since a chemotaxonomic analysis is still missing for a complete description, we determined the patterns of cellular fatty acids and polar lipids in this strain and compared them with *A. malicum* DSM 4132^T^. The composition of cellular fatty acids shown in Table 2 reveals some significant differences between the two strains. In particular, the content of dimethyl acetals derived from plasmalogens (alkenyl-acylphospholipids) is considerably higher in *A. malicum*. On the other hand, the proportion of unsaturated fatty acids is higher in ‘*A. dehalogenans*’. The patterns of polar lipids are also significantly different in the two strains (Table 3). The dominant polar lipid in *A. malicum* DSM 4132^T^ is diphosphatidylglycerol along with small amounts of phosphatidylserine, glycophospholipid and two unidentified lipids. In contrast, phosphatidylglycerol, phosphatidylcholine and an unidentified lipid were detected in ‘*A. dehalogenans*’.

**Table 2.** Cellular fatty acid composition of strain ‘*A. dehalogenans*’ DSM11527^T^ compared to the closely related type strain *A. malicum* DSM 4132^T^. Values are percentages of total fatty acids. Major fatty acids (>5% of the total amount) are given in bold; fatty acids that were detected only in trace amounts (<1.0% of the total amount) in all samples are not shown

Fatty acid	DSM 11527**^T^**	DSM 4132**^T^**
**C_14 : 1_* c*7**	tr	1.0
**C_14 : 0_**	tr	1.7
**C_16 : 0_ ALDE**	–	1.2
**C_16 : 1_* c*9**	**8.1**	**7.5**
**C_16 : 1_* c*11**	**8.4**	4.4
**C_16 : 0_**	**21.3**	**32.3**
**C_16 : 1_* c*9 DMA**	–	1.1
**C_16 : 0_ DMA**	–	**7.2**
**C_17 : 1_* c*9**	1.8	tr
**C_17 : 1_* c*11**	**11.0**	3.7
**C_17 : 0_**	4.5	2.2
**C_17 : 0_ DMA**	tr	1.3
**C_18 : 1_* c*11**	**24.9**	**13.0**
**C_18 : 1_* c*13**	**5.4**	1.5
**C_18 : 0_**	**7.9**	**9.8**
**C_18 : 1_* c*11 DMA**	–	1.7
**C_18 : 0_ DMA**	–	2.3

–, not detected; ALDE, aldehyde; *c*, *cis* isomer; DMA, dimethyl acetal; TR, trace amounts (<1.0% of the total amount).

**Table 3.** Phenotypic characteristics of *A. malicum* and ‘*A. dehalogenans*’. Unless otherwise stated, the data of both species were taken from the literature [3,28, 29]

Characteristic	‘*A. dehalogenans*’	*A. malicum*
Type strain	MC	MuME1
Source	Sewage digester sludge	Freshwater mud
Temperature optimum	25	30
pH optimum	7.3–7.7	7.5–8.0
*Substrates (+ CO_2_)*		
H_2_	+	+
CO	+	nd
CH_3_Cl	+	nd
CH_3_OH	+	−
Glycerol	+	+
Formate	w	w
Pyruvate	+	+
Lactate	+	+
Malate	+*	+
Fumarate	−	−
Methoxyacetate	−	−
Syingate	+	nd
Vanillate	+	nd
Trimethoxybenzoate	nd	+
Trimethoxycinnamate	nd	+
Fructose	+	+
Glucose	+	−
Major cellular fatty acids (>10% of total)*	C_18 : 1_* c*11, C_16 : 0_, C_17 : 1_* c*11	C_16 : 0_, C_18 : 1_* c*11
Polar lipids*	PG, PC, L	DPG, PS, GPL, L
Respiratory lipoquinones	−	nd
Cytochromes	−	−

*Results obtained in this study.

−, negative; +, positive; DPG, diphosphatidylglycerol; GPL, glycophospholipid; L, unidentified lipids; ND, no data; PG, phosphatidylglycerol; PS, phosphatidylserine; w, weakly positive.

The genus *Acetobacterium* belongs to the type of acetogen characterized by a lack of cytochromes and respiratory lipoquinones [28,29], so these compounds were not analysed.

Phenotypic differences between the two strains were also recognizable in the optimum growth temperature and substrate utilization pattern (Table 3). In contrast to the type strain of *A. malicum* MuME1^T^, ‘*A. dehalogenans*’ MC^T^ can grow on methanol and glucose. However, both strains can use malate as substrate, which is a distinguishing feature of the species *A. malicum* compared to the other established species of this genus.

In conclusion, the available genomic and phenotypic data justify the classification of strain MC^T^ as a novel subspecies of *A. malicum*, for which the name *A. malicum* subsp. *dehalogenans* subsp. nov. is proposed. The description of this subspecies automatically leads to the creation of the subspecies *A. malicum* subsp. *malicum* subsp. nov. represented by the type strain MuME1^T^.

### Emended description of *Acetobacterium malicum*

*Acetobacterium malicum* (ma’li.cum. N.L. neut. adj. *malicum*, pertaining to malic acid).

The following characteristics emend the original description by Tanaka and Pfennig [28]. The main components of the cellular fatty acid pattern are C_16 : 0_, C_18 : 1_* c*11, C_18 : 0_ and C_16 : 1_* c*9.

The size of the chromosome is in the range of 4.04–4.07 Mbp, and the G+C content of the genomic DNA is 43.7–43.8 mol%.

Strains of this species have been isolated from anoxic freshwater mud and sewage digester sludge. The type strain is MuME1^T^ (=DSM 4132^T^=ATCC 51201^T^). The GenBank/EMBL/DDBJ accession number for the 16S rRNA gene sequence of strain DSM 4132^T^ is X96957. A draft genome sequence is available under the accession number WJBE00000000.

## Description of *Acetobacterium malicum* subsp. *malicum* subsp. nov.

*Acetobacterium malicum* subsp. *malicum* (ma’li.cum. N.L. neut. adj. *malicum*, pertaining to malic acid).

The description of this subspecies follows the species description of *A. malicum* with the following additions that allow a differentiation from the subspecies *A. malicum* subsp. *dehalogenans*.

The optimum temperature for growth is 30 °C. The substrates methanol and glucose are not utilized. The cellular fatty acid pattern is lacking dimethyl acetals derived from plasmalogens. The dominating fatty acid is C_18 : 1_* c*11. The main identified polar lipid is diphosphatidylglycerol along with small amounts of phosphatidylserine and an unidentified glycophospholipid.

The size of the chromosome is 4.07 Mbp, and the G+C content of the genomic DNA is 43.7 mol%.

Strains of this species have been isolated from anoxic freshwater mud. The type strain is MuME1^T^ (=DSM 4132^T^=ATCC 51201^T^). The GenBank/EMBL/DDBJ accession number for the 16S rRNA gene sequence of strain DSM 4132^T^ is X96957. A draft genome sequence is available under the accession number WJBE00000000.

## Description of *Acetobacterium malicum* subsp. *dehalogenans* subsp. nov.

*Acetobacterium malicum* subsp. *dehalogenans* subsp. nov. (de.ha.lo'ge.nans. N.L. part. adj. *dehalogenans*, dehalogenating).

The definition of this subspecies is based on the descriptions of *A. malicum* and ‘*A. dehalogenans*’. The following characteristics allow a differentiation from the subspecies *A. malicum* subsp. *malicum*.

The optimum temperature for growth is 25 °C. The substrates methyl chloride, methanol and glucose are utilized for growth. The dominating fatty acid is C_16 : 0_. In addition to other DMAs, C_16 : 0_ DMA was found particularly. The main identified polar lipids are phosphatidylglycerol and phosphatidylcholine.

The size of the chromosome is 4.04 Mbp, and the G+C content of the genomic DNA is 43.8 mol%.

The type strain is MC^T^ (=DSM 11527^T^=NBRC 117038^T^) which has been isolated from sewage digester sludge. A complete genome sequence of strain DSM 11527^T^ is available under the accession number CP174122.

## Supplementary material

10.1099/ijsem.0.006783 Uncited Fig. S1.

## Abbreviations

ANI: average nucleotide identity
DCM: dichloromethane
dDDH: digital DNA–DNA hybridization
HGT: horizontal gene transfer
