# Supplementary material for: Unexpected 16S rRNA heterogeneity in ‘Acetobacterium dehalogenans’ and reclassification as Acetobacterium malicum subsp. dehalogenans subsp. nov
Source: Int J Syst Evol Microbiol. 2025 May 8;75(5):006783. doi: 10.1099/ijsem.0.006783 (PMC12062537; doi:10.1099/ijsem.0.006783)
Supplement: Uncited Fig. S1. [file ijsem-75-06783-s001.pdf]

International Journal of Systematic and Evolutionary Microbiology
Supplementary Material

Unexpected 16S rRNA heterogeneity in "*Acetobacterium dehalogenans*" and reclassification as *Acetobacterium malicum* subsp. *dehalogenans* subsp. nov.

Stefan Spring[1*], Jacqueline Wolf[2], Sarah Kirstein[2], Cathrin Spröer[3] and Boyke Bunk[3]

[1] Department Microorganisms, Leibniz Institute DSMZ - German Collection of Microorganisms and Cell Cultures, Braunschweig, Germany

[2] Department of Metabolomics and Services, Leibniz Institute DSMZ - German Collection of Microorganisms and Cell Cultures, Braunschweig, Germany

[3] Department Bioinformatics, Leibniz Institute DSMZ - German Collection of Microorganisms and Cell Cultures, Braunschweig, Germany

*** Correspondence:** Stefan Spring, stefan.spring@dsmz.de

The following PDF contains a supplementary figure.

Supplementary Fig. S1. Heatmap of 16S rRNA gene identity values obtained by pairwise comparison among genome-sequenced strains of the genus *Acetobacterium*. The green line marks areas containing 16S rRNA gene sequences representing a distinct species. The following species are represented: *A. carbinolicum* (DSM 2925^T, DSM 16427, KB-1), "*A. dehalogenans*" (DSM 11527^T), *A. woodii* (DSM 1030^T), *A. wieringae* (Y).

16S rRNA gene	1	2	3	4	5	6	7	8	9	10	11	12	13	14	15	16	17	18	19	20	21	22	23	24	25	26	27	28	29	30
**1. DSM 2925^T *rrsA***	100	99.93	99.87	99.80	99.67	99.87	99.93	99.93	99.54	99.48	99.21	99.21	99.21	99.21	99.21	98.37	99.02	98.36	99.34	98.56	98.55	98.35	98.41	98.28	98.28	97.71	97.71	97.71	97.71	98.55
**2. DSM 2925^T *rrsB***	99.93	100	99.93	99.87	99.47	99.80	100	100	99.48	99.54	99.28	99.28	99.28	99.28	99.28	98.30	98.95	98.30	99.28	98.49	98.48	98.28	98.35	98.22	98.22	97.65	97.65	97.65	97.65	98.49
**3. DSM 2925^T *rrsC***	99.87	99.93	100	99.80	99.67	99.74	99.93	99.93	99.41	99.48	99.21	99.21	99.21	99.21	99.21	98.24	98.89	98.23	99.21	98.43	98.55	98.35	98.41	98.28	98.28	97.58	97.58	97.58	97.58	98.42
**4. DSM 2925^T *rrsD***	99.80	99.87	99.80	100	99.61	99.93	99.87	99.87	99.34	99.41	99.41	99.41	99.28	99.41	99.41	98.43	99.08	98.43	99.41	98.62	98.35	98.15	98.22	98.08	98.08	97.51	97.51	97.51	97.51	98.36
**5. DSM 2925^T *rrsE***	99.67	99.47	99.67	99.61	100	99.54	99.74	99.74	99.34	99.41	99.21	99.21	99.21	99.21	99.21	98.30	98.69	98.03	99.28	98.23	98.22	98.15	98.08	98.08	97.95	97.51	97.51	97.51	97.51	98.36
**6. DSM 16427 *rrsA***	99.87	99.80	99.74	99.93	99.54	100	99.80	99.80	99.41	99.34	99.34	99.34	99.21	99.34	99.34	98.50	99.15	98.49	99.48	98.69	98.41	98.22	98.28	98.15	98.15	97.58	97.58	97.58	97.58	98.42
**7. DSM 16427 *rrsB***	99.93	100	99.93	99.87	99.74	99.80	100	100	99.48	99.54	99.28	99.28	99.28	99.28	99.28	98.30	98.95	98.30	99.28	98.49	98.48	98.28	98.35	98.22	98.22	97.65	97.65	97.65	97.65	98.49
**8. DSM 16427 *rrsC***	99.93	100	99.93	99.87	99.74	99.80	100	100	99.48	99.54	99.28	99.28	99.28	99.28	99.28	98.30	98.95	98.30	99.28	98.49	98.48	98.28	98.35	98.22	98.22	97.65	97.65	97.65	97.65	98.49
**9. DSM 16427 *rrsD***	99.54	99.48	99.41	99.34	99.34	99.41	99.48	99.48	100	99.80	99.21	99.21	99.21	99.21	99.21	98.04	98.56	98.17	99.02	98.10	98.08	97.89	97.95	97.82	97.82	97.38	97.38	97.38	97.38	98.23
**10. DSM 16427 *rrsE***	99.48	99.54	99.48	99.41	99.41	99.34	99.54	99.54	99.80	100	99.21	99.21	99.21	99.21	99.21	97.98	98.49	98.10	98.95	98.03	98.02	97.95	97.89	97.89	97.75	97.19	97.19	97.19	97.19	98.03
**11. KB-1 *rrsA***	99.21	99.28	99.21	99.41	99.21	99.34	99.28	99.28	99.21	99.21	100	100	99.87	100	100	97.91	98.49	97.96	98.88	98.03	97.75	97.56	97.62	97.49	97.49	96.99	96.99	96.99	96.99	97.83
**12. KB-1 *rrsB***	99.21	99.28	99.21	99.41	99.21	99.34	99.28	99.28	99.21	99.21	100	100	99.87	100	100	97.91	98.49	97.96	98.88	98.03	97.75	97.56	97.62	97.49	97.49	96.99	96.99	96.99	96.99	97.83
**13. KB-1 *rrsC***	99.21	99.28	99.21	99.28	99.21	99.21	99.28	99.28	99.21	99.21	99.87	99.87	100	99.87	99.87	97.77	98.36	97.83	98.75	97.90	97.75	97.56	97.62	97.49	97.49	96.99	96.99	96.99	96.99	97.83
**14. KB-1 *rrsD***	99.21	99.28	99.21	99.41	99.21	99.34	99.28	99.28	99.21	99.21	100	100	99.87	100	100	97.91	98.49	97.96	98.88	98.03	97.75	97.56	97.62	97.49	97.49	96.99	96.99	96.99	96.99	97.83
**15. KB-1 *rrsE***	99.21	99.28	99.21	99.41	99.21	99.34	99.28	99.28	99.21	99.21	100	100	99.87	100	100	97.91	98.49	97.96	98.88	98.03	97.75	97.56	97.62	97.49	97.49	96.99	96.99	96.99	96.99	97.83
**16. DSM 11527^T *rrsA***	98.37	98.30	98.24	98.43	98.30	98.50	98.30	98.30	98.04	97.98	97.91	97.91	97.77	97.91	97.91	100	99.35	98.56	99.02	98.63	98.68	98.88	98.88	99.01	98.74	98.69	98.69	98.69	98.69	98.62
**17. DSM 11527^T *rrsB***	99.02	98.95	98.89	99.08	98.69	99.15	98.95	98.95	98.56	98.49	98.49	98.49	98.36	98.49	98.49	99.35	100	99.21	99.41	99.28	98.35	98.35	98.48	98.48	98.35	98.30	98.30	98.30	98.30	99.14
**18. DSM 11527^T *rrsC***	98.36	98.30	98.23	98.43	98.03	98.49	98.30	98.30	98.17	98.10	97.96	97.96	97.83	97.96	97.96	98.56	99.21	100	98.62	99.80	97.69	97.69	97.76	97.76	97.82	98.75	98.75	98.75	98.75	99.34
**19. DSM 11527^T *rrsD***	99.34	99.28	99.21	99.41	99.28	99.48	99.28	99.28	99.02	98.95	98.88	98.88	98.75	98.88	98.88	99.02	99.41	98.62	100	98.82	97.89	98.02	98.02	98.15	97.89	97.84	97.84	97.84	97.84	98.68
**20. DSM 11527^T *rrsE***	98.56	98.49	98.43	98.62	98.23	98.69	98.49	98.49	98.10	98.03	98.03	98.03	97.90	98.03	98.03	98.63	99.28	99.80	98.82	100	97.89	97.89	97.95	97.95	98.02	98.82	98.82	98.82	98.82	99.41
**21. DSM 1030^T *rrsA***	98.55	98.48	98.55	98.35	98.22	98.41	98.48	98.48	98.08	98.02	97.75	97.75	97.75	97.75	97.75	98.68	98.35	97.69	97.89	97.89	100	99.74	99.80	99.67	99.67	98.28	98.28	98.28	98.28	97.89
**22. DSM 1030^T *rrsB***	98.35	98.28	98.35	98.15	98.15	98.22	98.28	98.28	97.89	97.95	97.56	97.56	97.56	97.56	97.56	98.88	98.35	97.69	98.02	97.89	99.74	100	99.87	99.87	99.74	98.28	98.28	98.28	98.28	97.96
**23. DSM 1030^T *rrsC***	98.41	98.35	98.41	98.22	98.08	98.28	98.35	98.35	97.95	97.89	97.62	97.62	97.62	97.62	97.62	98.88	98.48	97.76	98.02	97.95	99.80	99.87	100	99.87	99.74	98.35	98.35	98.35	98.35	98.02
**24. DSM 1030^T *rrsD***	98.28	98.22	98.28	98.08	98.08	98.15	98.22	98.22	97.82	97.89	97.49	97.49	97.49	97.49	97.49	99.01	98.48	97.76	98.15	97.95	99.67	99.87	99.87	100	99.74	98.35	98.35	98.35	98.35	98.02
**25. DSM 1030^T *rrsE***	98.28	98.22	98.28	98.08	97.95	98.15	98.22	98.22	97.82	97.75	97.49	97.49	97.49	97.49	97.49	98.74	98.35	97.82	97.89	98.02	99.67	99.74	99.74	99.74	100	98.35	98.35	98.35	98.35	98.02
**26. Y *rrsA***	97.71	97.65	97.58	97.51	97.51	97.58	97.65	97.65	97.38	97.19	96.99	96.99	96.99	96.99	96.99	98.69	98.30	98.75	97.84	98.82	98.28	98.28	98.35	98.35	98.35	100	100	100	100	99.15
**27. Y *rrsB***	97.71	97.65	97.58	97.51	97.51	97.58	97.65	97.65	97.38	97.19	96.99	96.99	96.99	96.99	96.99	98.69	98.30	98.75	97.84	98.82	98.28	98.28	98.35	98.35	98.35	100	100	100	100	99.15
**28. Y *rrsC***	97.71	97.65	97.58	97.51	97.51	97.58	97.65	97.65	97.38	97.19	96.99	96.99	96.99	96.99	96.99	98.69	98.30	98.75	97.84	98.82	98.28	98.28	98.35	98.35	98.35	100	100	100	100	99.15
**29. Y *rrsD***	97.71	97.65	97.58	97.51	97.51	97.58	97.65	97.65	97.38	97.19	96.99	96.99	96.99	96.99	96.99	98.69	98.30	98.75	97.84	98.82	98.28	98.28	98.35	98.35	98.35	100	100	100	100	99.15
**30. Y *rrsE***	98.55	98.49	98.42	98.36	98.36	98.42	98.49	98.49	98.23	98.03	97.83	97.83	97.83	97.83	97.83	98.62	99.14	99.34	98.68	99.41	97.89	97.96	98.02	98.02	98.02	99.15	99.15	99.15	99.15	100

100% **99.5%** **99.0%** **98.5%** **98.0%** **97.5%** **97.0%**
